# Cell Clearing Systems as Targets of Polyphenols in Viral Infections: Potential Implications for COVID-19 Pathogenesis

**DOI:** 10.3390/antiox9111105

**Published:** 2020-11-10

**Authors:** Fiona Limanaqi, Carla Letizia Busceti, Francesca Biagioni, Gloria Lazzeri, Maurizio Forte, Sonia Schiavon, Sebastiano Sciarretta, Giacomo Frati, Francesco Fornai

**Affiliations:** 1 Department of Translational Research and New Technologies in Medicine and Surgery, University of Pisa, Via Roma 55, 56126 Pisa, Italy; f.limanaqi@studenti.unipi.it (F.L.); gloria.lazzeri@unipi.it (G.L.); 2 I.R.C.C.S. Neuromed Pozzilli, Via Atinense, 18, 86077 Pozzilli, Italyfrancesca.biagioni@neuromed.it (F.B.); maurizio.forte@neuromed.it (M.F.); sebastiano.sciarretta@uniroma1.it (S.S.); fraticello@inwind.it (G.F.); 3 Department of Medico-Surgical Sciences and Biotechnologies, Sapienza University of Rome, Corso della Repubblica 79, 40100 Latina, Italy; sonia.schiavon@uniroma1.it

**Keywords:** autophagy, immunoproteasome, coronavirus, inflammation, resveratrol, quercetin, kaempferol, baicalin, cordycepin

## Abstract

The novel coronavirus named severe acute respiratory syndrome coronavirus 2 (SARS-CoV-2) has generated the ongoing coronavirus disease-2019 (COVID-19) pandemic, still with an uncertain outcome. Besides pneumonia and acute lung injury (ALI) or acute respiratory distress syndrome (ARDS), other features became evident in the context of COVID-19. These includes endothelial and coagulation dysfunction with disseminated intravascular coagulation (DIC), and multiple organ dysfunction syndrome (MODS), along with the occurrence of neurological alterations. The multi-system nature of such viral infection is a witness to the exploitation and impairment of ubiquitous subcellular and metabolic pathways for the sake of its life-cycle, ranging from host cell invasion, replication, transmission, up to a cytopathic effect and overt systemic inflammation. In this frame, alterations in cell-clearing systems of the host are emerging as a hallmark in the pathogenesis of various respiratory viruses, including SARS-CoV-2. Indeed, exploitation of the autophagy and proteasome pathways might contribute not only to the replication of the virus at the site of infection but also to the spreading of either mature virions or inflammatory mediators at both cellular and multisystem levels. In this frame, besides a pharmacological therapy, many researchers are wondering if some non-pharmacological substances might counteract or positively modulate the course of the infection. The pharmacological properties of natural compounds have gained increasing attention in the field of alternative and adjunct therapeutic approaches to several diseases. In particular, several naturally-occurring herbal compounds (mostly polyphenols) are reported to produce widespread antiviral, anti-inflammatory, and anti-oxidant effects while acting as autophagy and (immuno)-proteasome modulators. This article attempts to bridge the perturbation of autophagy and proteasome pathways with the potentially beneficial effects of specific phytochemicals and flavonoids in viral infections, with a focus on the multisystem SARS-CoV-2 infection.

## 1. Introduction

The coronavirus disease 2019 (COVID-19), generated by the novel coronavirus named SARS-CoV-2, emerged as a rapidly spreading communicable disease, still with an uncertain outcome worldwide. Similar to other respiratory viruses, including influenza viruses and the two other members of the *Coronaviridae* family SARS-CoV and MERS-CoV, SARS-CoV-2 produces multifaceted, often severe effects in the human body [1,2]. A considerable number of patients affected by COVID-19 develops severe respiratory complications due to interstitial pneumonia and acute lung injury (ALI)/ acute respiratory distress syndrome (ARDS) with lung vasculitis and respiratory failure, which are associated with a high mortality rate [2,3]. The respiratory symptoms caused by SARS-CoV-2 are similar to that of influenza virus, SARS-CoV, and MERS-CoV infection, being associated with rapid lung inflammation and overt cytokine storm [1,3,4]. Besides pneumonia and ARDS/ ALI, multiple-organ damage along with the occurrence of neurological alterations, have become more and more evident in the context of COVID-19 [5,6,7,8,9,10]. As documented by autopsy studies, the vascular insult is devastating even within the lung itself, and a marked inflammatory response within blood vessels is observed [5,8]. COVID-19 symptoms also include cardiovascular events, such as stroke, multiple thromboembolism, as well as acute myocarditis, and myocardial infarction, even in the absence of lung involvement [6,11,12,13,14,15]. SARS-CoV-2 might trigger an exacerbated immunological response and cytokine storm, leading to a “viral sepsis” [16], which can indirectly contribute to cardiovascular injury and might explain the causes of cardiac and vascular damage. Nonetheless, similar to other coronaviruses (CoVs), SARS-CoV-2 might also directly infect cardiomyocytes, endothelial cells, and pericytes, thereby causing progressive myocardial damage and vasculitis characterized by extensive thrombosis [16,17,18]. In either case, the severe involvement of blood vessels is expected to spread the disease within various organs, preferring those with a high blood supply. Either a direct viral infection or a cytokine storm spreading through a synapse-connected route might also explain the central nervous system (CNS) alterations that are observed during SARS-CoV-2 infection [19,20,21]. SARS-CoV-2 was detected in the cerebrospinal fluid and also within neuronal and endothelial brain cells of COVID-19 patients [19,20]. The neuro-invasive potential of SARS-CoV-2 is not fully characterized, though it appears reminiscent of that of SARS-CoV [21,22,23]. In detail, SARS-CoV produces a massive infection of the brainstem in both patients and experimental animals [24,25]. In light of the high similarity with SARS-CoV, it is expected that SARS-CoV-2 produces similar effects. The kind of neurological symptoms reported in COVID-19 patients [9,10] appears to be compatible with this scenario. As reported for SARS-CoV, the involvement of a few neurons within the ventrolateral medulla, which generates the cardiorespiratory rhythm, might affect the cardiovascular and respiratory systems, even following small focal damage of the brainstem [24,25]. In this way, the CNS, which provides profuse innervation to multiple organs including the lung, the heart, and blood vessels themselves, might contribute to generating and sustaining the multi-system disorder associated with SARS-CoV-2 infection.

This is compatible with the specific yet widespread placement of the angiotensin-converting enzyme type 2 (ACE2), which represents the gateway for SARS-CoV-2 entry within infected cells. In fact, ACE2 is abundantly expressed in the lungs, heart, kidney, vasculature, and cardiorespiratory neurons within the brainstem [18,26,27]. The ACE2 receptor might provide the opportunity to the virus for multi-organ infectivity, resulting in multiple organ dysfunction syndrome (MODS), through impairment of ubiquitous subcellular structures and metabolic machinery to complete its life-cycle and transmission.

Despite controversial results, the occurrence of alterations in cell-clearing pathways is emerging as a hallmark in the pathogenesis of numerous respiratory viruses, including influenza virus, MERS-CoV, SARS-CoV, and SARS-CoV-2 [28,29,30,31,32,33,34]. This is the case of the ubiquitin-proteasome system (UPS) and autophagy, which recently attracted much attention as potential therapeutic targets in COVID-19 pathogenesis [30,31,32,34,35,36,37]. Within eukaryotic cells, autophagy and the UPS operate coordinately, to clear intracellular damaged proteins, organelles, and also bacterial and viral pathogens [38,39]. Autophagy and the UPS are also promiscuously implicated in innate and adaptive immunity, by modulating constitutive inflammatory responses while ensuring antigen processing and plasma membrane presentation to CD4+ and CD8+ T-cells via MHC-II and MHC-I molecules, respectively [39,40,41,42,43]. In this scenario, autophagy and proteasome configure as sentinels in the mechanisms underlying cell-to-cell, and multi-system communication in both health and disease [39,44]. Both autophagy and UPS are commonly altered in several inflammatory-related conditions, including viral infections as well as pulmonary, metabolic, cardiovascular, and neurological diseases [30,33,34,38,41,44,45,46,47,48]. Thus, understanding how SARS-CoV-2 alters these pathways, might contribute to disclosing the specific failure that occurs within different cell types, reflecting the multi-organ/multi-system nature of COVID-19.

Identifying new targets and drugs against SARS-CoV-2 represents a rush against time since no therapies are shown to be fully effective and devoid of adverse effects [49]. The need for natural-based autophagy modulators was recently emphasized in the frame of COVID-19 pathogenesis [31,35]. In this context, the effects of naturally-derived compounds have become an area of immense interest, due to their multi-system-multi-target, mostly anti-inflammatory and anti-oxidant effects, coupled with a relatively safe toxicity profile at potentially therapeutic doses [50,51,52,53,54,55]. In detail, several naturally occurring compounds (mostly polyphenols) from traditional herbal medicine (THM), are reported to produce beneficial effects in various inflammatory disorders, including viral infections, as well as respiratory, cardiovascular, and neurological diseases [55,56,57,58,59,60,61,62,63,64,65,66,67]. Remarkably, the main bioactive compounds of these formulas, such as resveratrol, quercetin, kaempferol, baicalin, and cordycepin, while counteracting viral replication and abnormally activated inflammatory/apoptotic pathways, do act as autophagy or UPS modulators as well [57,67,68,69,70,71,72,73].

The present review focuses on the potential link between the effects of specific phytochemicals and the tuning of autophagy and UPS pathways in the frame of the multi-system disorder produced by CoV infections. The article covers autophagy/UPS-related and phytochemical-targeted molecular pathways that are potentially implicated in the SARS CoV-2 viral gateways, its fruitful replication within host cells, inflammatory and oxidative alterations affecting cell function and viability, and eventually, the extracellular spreading of the virus and viral-induced inflammatory mediators. Dissecting the molecular mechanisms through which autophagy/UPS-modulating phytochemicals might provide beneficial effects in respiratory viral infections and inflammatory-related conditions might contribute to disclosing potential targets and hopefully, to developing prophylactic or adjunct therapeutic strategies in multisystem COVID-19.

## 2. What We Know, Do Not Know, and What We Can Hypothesize on the Role of Autophagy and Proteasome in Viral Infections and COVID-19 Pathogenesis

### 2.1. Autophagy, Viral Infection, and Inflammation

Autophagy is an evolutionarily-conserved intracellular mechanism that mediates degradation of misfolded proteins, intracellular aggregates, and damaged organelles through a complex degradative process that plays a crucial role in eukaryotic cell homeostasis. Autophagy is activated in response to cellular stresses, including starvation, endoplasmic reticulum (ER) stress, and oxidative stress, thereby limiting or modulating cell death. Accordingly, several cytoplasmic elements such as proteins, lipids, sugars, nucleic acids, senescent or damaged organelles, or pathogens, are isolated and enclosed into a double-membrane nascent vacuole called phagophore, which then matures to seal in the autophagosome. Autophagy machinery might be initiated either as a non-selective process or involve several adaptor/receptor proteins such as SQSTM1/p62, which deliver ubiquitinated cargoes, and also the proteasome itself, to the forming autophagosome [48,54]. P62 represents an autophagy substrate that is widely hired as a marker of autophagy flux progression, which follows up the fusion of autophagosomes/amphisomes with lysosomes. In fact, the mature autophagosome fuses with endosomes and multivesicular bodies (MVBs) giving origin to the amphisome, which eventually fuses with the lysosome that provides acidic hydrolases for substrate breakdown. Once engulfed within the autolysosome, the cargo is degraded, while some metabolic by-products are recycled and reintroduced in cellular metabolism. Different nutrient-sensing pathways convey signals to the autophagy machinery by recruiting autophagy-related (ATG) proteins to orchestrate the fine steps of autophagy development, starting from the biogenesis and maturation of autophagosomes, up to the fusion with endosomes and lysosomes [48,54,74]. In response to the metabolic needs of the cells, the concerted actions of the phosphatidylinositol 3-kinase (PI3K)/AKT and the Unc-51 like autophagy activating kinase-1 (ATG1/ULK1) complexes are key to promote autophagy initiation through nucleation of phagophores [74]. As a master nutrient sensor, the mammalian target of rapamycin complex 1 (mTORC1) acts as a primary regulator of autophagy initiation in mammalian cells through phosphorylation of ATG13 and inhibition of ATG1/ULK1. mTORC1-dependent inhibition and 5′ AMP-activated Protein Kinase (AMPK)-mediated activation of the ULK1 complex are the most characterized pathways that couple nutrient sensing to autophagosome biogenesis [48,54,74]. Phosphorylation of the PI3K-III regulatory subunit Beclin-1 (ATG6, BECN1), the formation of the BECN1/VPS34/ATG14 complex, and phosphorylation of the activating molecule in BECN1-regulated autophagy (AMBRA1) by ULK1, are equally important in finely tuning the autophagy process from phagosome biogenesis up to fusion with lysosomes, while balancing autophagy with p38MAPK- and ERK/JNK-related apoptosis [74]. Conversion of ATG8 [microtubule-associated protein light chain 3 (LC3) in mammals] into soluble LC3I, ubiquitination-like enzymatic lipidation of LC3I to form lipid-bound LC3II isoform, and finally the incorporation of LC3II into the phagophore membrane are key steps for the vacuole to expand and seal, thus, allowing the cytoplasmic elements to be properly engulfed [54].

Along with mTOR- and AMPK-related autophagy, activation of the NAD-dependent deacetylase Sirtuin-1 (SIRT1) promotes autophagy by de-acetylating different substrates, including ATG5, ATG7, LC3, as well as transcription factor forkhead box O3 (FOXO3), which transcriptionally up-regulates several pro-autophagic genes [54,75]. Finally, the transcription factor EB (TFEB) also promotes autophagy by activating the genes involved in autophagy, autophagosome-lysosome fusion, and lysosomal biogenesis [48,54,76].

**Figure 1:**
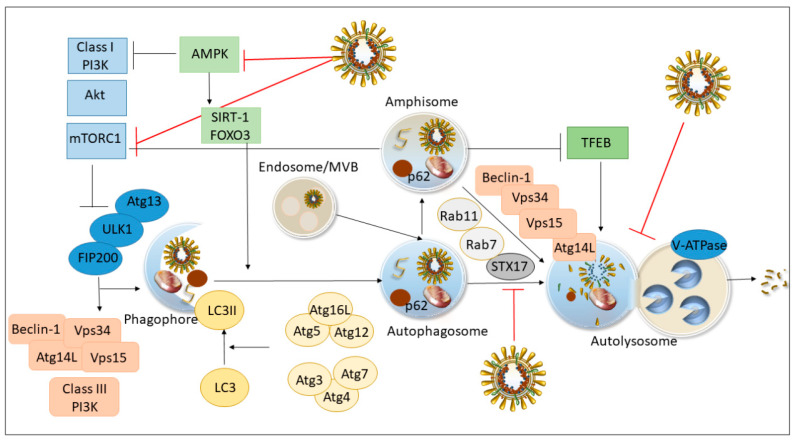
Molecular steps of the autophagy pathway and viral-targeted events hampering autophagy progression. The class I phosphatidylinositol 3-kinase (PI3K)/AKT/mammalian target of rapamycin complex 1 (mTORC1), and 5′ AMP-activated Protein Kinase (AMPK) pathways orchestrate autophagy initiation through regulation of kinase-1 (Atg1/ULK1) and Beclin-1/Vps34/Vps15/Atg14 complexes. mTORC1 inhibits while AMPK promotes autophagy initiation. The Beclin-1/Vps34/Vps15/Atg14 complex, the NAD-dependent deacetylase Sirtuin-1 (SIRT1), and the transcription factors forkhead box O3 (FOXO3) and EB (TFEB) promote several steps of the autophagy process, from phagophore biogenesis up to the fusion of autophagosomes with lysosomes. Various ATG products ranging from Atg3 to Atg16L are involved in the conversion of LC3 into soluble LC3I, ubiquitination-like enzymatic lipidation of LC3I to form lipid-bound LC3II isoform, and finally the incorporation of LC3II into the phagophore membrane, which is a key for the vacuole to expand and seal. Along with ATG products, specific evolutionarily conserved multitasking proteins that regulate intracellular endosomal/secretory trafficking pathways, such as Rab11, Rab7, and Syntaxin 17 (STX17), are implicated in autophagosome maturation and autophagosome–lysosome fusion. The influenza virus blocks autophagic flux through activation of PI3K/AKT and downregulation of the autophagosome-lysosome fusion factors Syntaxin-17 (STX17) and V-type proton ATPase subunit (V-ATPase). MERS-CoV and SARS-CoV-2 lead to AKT activation, AMPK inhibition, and subsequent decrease in Beclin-1 and Atg14 levels, underlying the lack of fusion of autophagosomes with lysosomes. SARS-CoV-2 also downregulates mTOR, which is likely to enhance the availability of membrane precursors forming autophagy-like vesicles where the virus replicates.

Along with ATG products, specific evolutionary conserved multitasking proteins that regulate intracellular endosomal/secretory trafficking pathways, such as G-coupled Ras-related proteins in brain (Rab GTPases) and SNAREs (Soluble N-ethylmaleimide-sensitive factor attachment protein receptor), are implicated in autophagosome maturation and autophagosome-lysosome fusion [77]. Paradoxically, blockade of lysosomal function can lead to enhanced autophagy initiation via inhibition of mTORC1 [78]. This might be key to comprehend the effects of SARS-CoV-2 at the level of the autophagy machinery, as both reduced lysosomal degradation and autophagosome accumulation via mTOR-ULK1 signaling might facilitate viral replication and transmission (Figure 1) [30,31,78].

In detail, similar to SARS-CoV and MERS-CoV, SARS-CoV-2 is internalized upon the interaction of spike proteins with ACE2 [79]. Within host cells, the virus is first stored within the endosomal compartment, from where it releases the viral RNA to initiate replication. Translation and eventually packaging of mature virions occur within the ER and Golgi apparatus. Many viruses, including SARS-CoV-2, induce the accumulation of autophagosomes likely to promote their replication herewith [31,80]. In line with this, many studies have documented an early increase in the number of double-membrane, autophagy-like vacuoles and endosomes/MVBs, which at first suggested an abnormal activation of the autophagy pathway, during infection by either influenza viruses or CoVs [80,81,82,83,84].

Nonetheless, these concepts were recently challenged by accumulating evidence showing that autophagy compartments are not fully mature, neither do they merge with lysosomes. For instance, the influenza virus blocks autophagic flux through activation of PI3K/AKT and downregulation of the autophagosome–lysosome fusion factors Syntaxin-17 (STX17) and V-type proton ATPase subunit (Figure 1) [85,86]. Autophagy impairment during the influenza virus is due to either NS1 or M2 viral proteins, and it is associated with enhanced viral protein release and virus-induced apoptotic cell death [85,86,87]. Conversely, restoring autophagic flux counteracts these effects [86]. During influenza virus infection, the non-structural protein NS1 might also impede autophagosome formation through activation of the PI3K/AKT/mTOR pathway [88]. This is in line with evidence in MERS-CoV-infected hepatocytes showing an abnormal activation of the PI3K/AKT/mTOR and ERK/MAPK pathways, which are known to inhibit autophagy [89]. Supporting an impairment of autophagy, pretreatment with pharmacological inhibitors of these pathways inhibits MERS-CoV infection [89].

An impairment of autophagosome formation during influenza virus infection was also associated with the downregulation of the endocytic protein Rab11a (Figure 1) [88]. In detail, the loss of the endosomal protein Rab11a impairs the formation of autophagosomes and the engulfment of viral ribonucleoprotein (vRNP) complexes by autophagosomes in infected cells. This indicates that Rab11a-positive recycling endosomes, while serving as membrane donors for the phagophore elongation, also act as a receptor for the engulfment of vRNP complexes within autophagy compartments [88]. This adds to previous evidence indicating the ER as the major source of viral-induced autophagosomes, which is documented in the avian bronchitis virus infection [28,83]. Here, the non-structural replicase protein nsp6, which is also present in MERS-CoV and SARS-CoV-2, contributes to occluding the delivery of viral components to lysosomes, by impairing the expansion and maturation of ER-derived autophagosomes [28].

A defect in the autophagy flux due to impaired fusion of autophagosomes with lysosomes is confirmed in SARS-CoV and MERS-CoV, and it was recently documented for SARS-CoV-2 (Figure 1) [30,31,80].

Both MERS-CoV and SARS-CoV-2 infections lead to increased levels of phosphorylated AKT and subsequent decrease in BECN1 and ATG14 levels, underlying the lack of fusion of autophagosomes with lysosomes [30,31]. In fact, SARS-CoV-2 downregulates the autophagy-inducing polyamine spermidine and it occludes AMPK and BECN1-dependent autophagy, while exogenous administration of either spermidine, AKT inhibitors, and BECN1 stabilizing agents, inhibits SARS-CoV-2 propagation in vitro [31]. Paradoxically, the virus also induces downregulation of mTOR, indicating that the SARS-CoV-2-induced autophagy block might be in part independent of mTOR activity, at least during the early phases of infection. While eventually occluding the conventional autophagy-dependent degradation of viral contents, which is known as “virophagy/xenophagy”, a non-effective autophagic flux might also enhance the availability of ER-derived membrane precursors forming autophagy-like vesicles that are generated during viral replication [31,90]. In this scenario, the early evidence on a deleterious role of autophagy/endosomal pathway might also be a misinterpretation when focusing on single autophagy-related genes/proteins or pathways, rather than on the autophagy flux.

At the same time, SARS-CoV-2-induced reduction of mTOR-dependent glycolysis and protein translation [31] is compatible with the manipulation of the cell cycle or induction of apoptosis, which is used by many viruses as a strategy to promote their infection cycles [91,92,93,94]. This is also documented for influenza virus and SARS-CoV, which block G1/S phase transition and cell proliferation [91,94]. This occurs mostly through the downregulation of phosphorylated cell cycle regulator retinoblastoma (Rb), which is placed downstream of specific GTPases, transcription factors, and cyclin-dependent kinase modulators, such as the Ras homolog gene family member A (RhoA), p53, and p21. Since a mutual interplay exists between autophagy, UPS, and the above-mentioned [95], it would be worth investigating whether and how SARS-CoV-2 might exploit such a coordinated response between cell-clearing systems and cell-cycle arrest.

**Figure 2:**
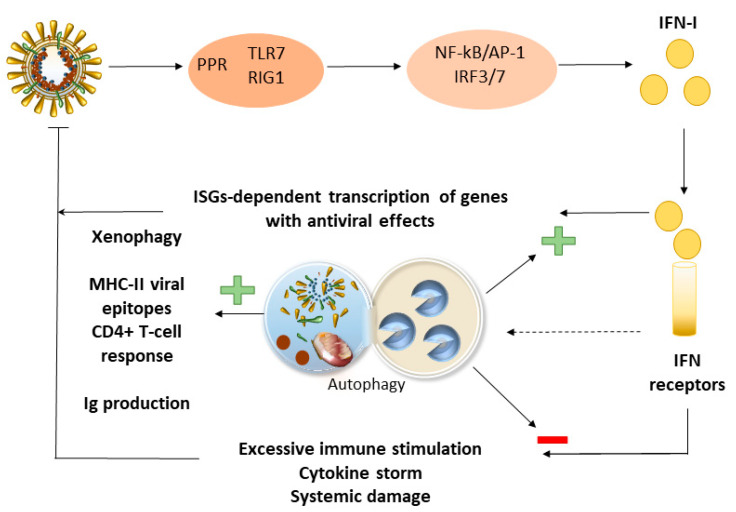
During viral infection, viral pathogen-associated molecular patterns (PAMPs) are detected by host cell pattern recognition receptors (PRRs). PRRs like TLR7 and retinoic acid-inducible gene-I (RIG-I) initiate antiviral responses through activation of transcriptional factors NF-kB/AP-1 and IRF3/7. This promotes the generation of IFN-α/β and other pro-inflammatory cytokines as a first response to the viral infection. Subsequently, IFN-α/β binds to its receptors, inducing the interferon-stimulated genes (ISGs)-dependent transcription of multiple genes with antiviral effects. Excessive immune activation and IFN production might cause damage to the body. In this frame, autophagy is key to balancing antimicrobial immune responses by inducing viral clearance (xenophagy), CD4+ T-cell-dependent responses, and immunoglobulin production, meanwhile preventing excessive inflammation and immune stimulation. By affecting the autophagy machinery, viruses might either hijack the host immune response or promote excessive immune stimulation and cytokine storm.

Autophagy also plays a balancing role intended to ensure a measured anti-inflammatory response, while avoiding excessive inflammatory tissue damage [41,96]. During viral infection, viral pathogen-associated molecular patterns (PAMPs) are detected by host cell pattern recognition receptors (PRRs). PRRs like TLR7 and retinoic acid-inducible gene-I (RIG-I) initiate antiviral responses by binding of IRAK kinase to MyD88 and phosphorylation of transcriptional factors (NF-kB/AP-1 and IRF3/7). Transcriptional factors promote the generation of IFN-α/β and other pro-inflammatory cytokines as a first response to the viral infection. Subsequently, IFN-α/β binds to its receptors, inducing the interferon-stimulated genes (ISGs)-dependent transcription of multiple genes with antiviral effects. Excessive immune activation and IFN production might cause damage to the body (Figure 2). Host cells have developed intricate approaches to balance the expression of IFN- α/β, with both proteasomes and autophagy involved in this regulation [96]. Autophagy can downregulate IFN production by the degradation of viral PAMPs associated with PRRs (e.g., TLR7) [96,97]. Association of RIG-I with negative factors leads to its degradation via either p62-mediated autophagy or the proteasome [98,99,100]. In turn, IFN binding to its receptors can induce the formation of both immunoproteosome and autophagy (xenophagy), which is involved in the degradation of viral products and induction of the antiviral state of the IFN-treated cells [96]. An autophagy impairment during CoV infection might occur along with alterations in IFN-type I signalling, which is key in the early phases of viral infection [96,101,102]. As shown in MERS-CoV, besides nsp6, the autophagy impairment is also associated with the non-structural viral proteins 4b and p5 [30], which act as antagonists of the IFN-type I signaling [103,104]. In influenza virus-infected cells, autophagosome formation and p62 accumulation witnessing a stall in autophagy progression, are accompanied by limited IFN-β expression, which might fuel viral replication and spread [105]. On the other hand, the interaction of viral proteins with BECN1 might contribute to increasing viral replication by hampering fusion of the autophagosome with the lysosome, meanwhile promoting excessive IFN production [106]. This is remarkable since CoVs impair BECN1-dependent autophagy flux [30,31], which in turn, is key to balancing antimicrobial immune responses by promoting xenophagy, while preventing excessive immune stimulation (Figure 2) [107].

The crosstalk between autophagy and IFN-I responses might serve as a major bridge linking autophagy to innate antiviral immunity, and many viruses can manipulate the key molecules of the autophagy process to alter IFN-I production [96]. However, a direct link between impairment of autophagy and IFN signalling remains to be confirmed for CoVs, as most viral proteins are multifunctional and interact with multiple independent cell processes.

Autophagy is also involved in the processing and (cross)-presentation of antigens on MHC-II molecules and subsequent activation of CD4+ T-cell-dependent response, while surveilling CD8+ T-cell-dependent cytotoxic response via degradation of MHC-I [41,107]. In addition to T-cell-mediated immunity, autophagy is also required for in vivo immunoglobulin production and secretion by plasma cells [108], which might be crucial for the resolution of COVID-19 pathogenesis [109]. On the other hand, autophagy is key to preventing oxidative damage and excessive inflammation, for instance by counteracting the COX2-NF-kB-NLRP3 pathways [41,110,111]. Rescuing autophagy might produce mitigation of oxidative damage and excessive inflammatory response in a variety of tissues and cells that are known to be affected during COVID-19, including lung epithelial cells, endothelial cells, cardiomyocytes, neurons, and glia [46,111,112,113,114,115]. In line with this, the belief that autophagy activation during coronavirus infection might contribute to disease pathogenesis is currently being challenged by the worsening in the severe respiratory stress upon administration of autophagy/lysosomal blockers [36,116]. Although such evidence remains contradictory and needs to be confirmed by rigorous scientific scrutiny, it seems to fit with the pro-inflammatory effects that are produced by either genetic or pharmacological autophagy blockade [46,117,118].

### 2.2. Immunoproteasome, Viral Infection, and Inflammation

Viral infections, and pro-inflammatory/oxidative stimuli in general, while potentially altering autophagy, recruits the immunoproteasome [119]. This represents an alternative, cytokine IFN-γ, and TNF-α-inducible UPS isoform that owns particular structural features and an increased chymotrypsin-like catalytic capacity compared to standard proteasomes [120,121]. These features enable immunoproteasome-dependent processing of either the self or viral antigen peptides and subsequent activation of CD8+ T-cell-dependent adaptive response via MHC-I presentation at the plasma membrane [44,119,120].

**Figure 3:**
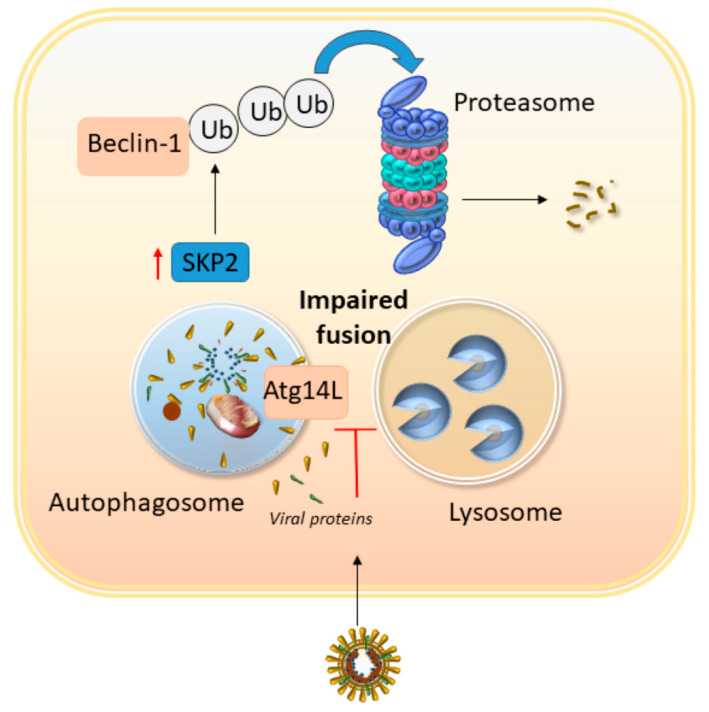
Abnormal UPS activity might contribute to impairing the autophagy machinery during CoVs infections through SKP2 recruitment and UPS-dependent degradation of BECN1.

As a first-line defense, the immunoproteasome is key to counteract viral replication within the infected cell, though its role appears context- and virus-dependent [119,122,123]. For instance, contrary to SARS-CoV, MERS-CoV infection in lung epithelial cells induces a massive downregulation of the host immunoproteasome subunits and MHC genes [122]. Although this was assessed only at the mRNA levels, which does not necessarily reflect a decrease in immunoproteasome catalytic activity, this might represent a virus-specific mechanism through which MERS-CoV attempts to hijack immune activation. This seems compatible with recent evidence that SARS-CoV-2 leads to an mTOR inhibition, which in turn, is reported to reduce immunoproteasome recruitment, despite increasing overall UPS-dependent protein degradation [124,125]. Abnormal UPS activity might contribute to impairing the autophagy machinery. For instance, MERS-CoV infection recruits the E3 ligase SKP2 to impair autophagy progression through the UPS-dependent degradation of BECN1 (Figure 3) [30]. Additionally, the immunoproteasome impairs autophagy through ATG5 and PTEN degradation, which contributes to exacerbating cardiac hypertrophy in mice models [126,127].

Although immunogenic epitopes in both the structural and non-structural proteins of SARS-CoV-2 that could interact with the MHC-I alleles were identified [128,129], the role of immunoproteasome in SARS-CoV-2 infection specifically remains to be investigated. While it might be seminal to elicit an anti-viral adaptive response in the site of infection, it is likely that during the late phases of infection, when a cytokine storm takes place, the immunoproteasome might be strongly and persistently recruited to promote an excessive immune reaction fueling autophagy impairment. This is supported by the fact that immunoproteasome recruitment occurs through activation of molecular pathways such as PKC, NF-kB, JAK/STAT, AKT/mTOR, ACE/Angiotensin-II/ATR1, and TLR/RAGEs, which are known to impinge on the autophagy machinery [39,44,48,125,126,127,130,131,132,133,134,135,136]. Additionally, an abnormal persistence of immunoproteasomes might perpetuate excessive inflammation and immune responses to promote acute respiratory distress, vasculitis, hypertension, myocarditis, and neuronal damage [44,123,131,132,137,138,139,140]. This is associated with abnormal cytotoxic CD8+ T-cell responses also involving neuronal cells, which under excessive pro-inflammatory conditions upregulate MHC-I molecules, just like antigen-presenting cells [141]. In fact, immunoproteasome recruitment along with concomitant impairment of autophagy flux is described during inflammation and oxidative damage occurring in neurodegeneration and neurotoxicity, including that associated with viral infections [44,142,143]. In this context, autophagy finely-tunes the immunoproteasome-dependent CD8+ T-cell responses by handling the turnover of MHC-I molecules [107], which suggests that an autophagy impairment might contribute to exacerbating immunoproteasome-dependent cytotoxic response.

**Figure 4:**
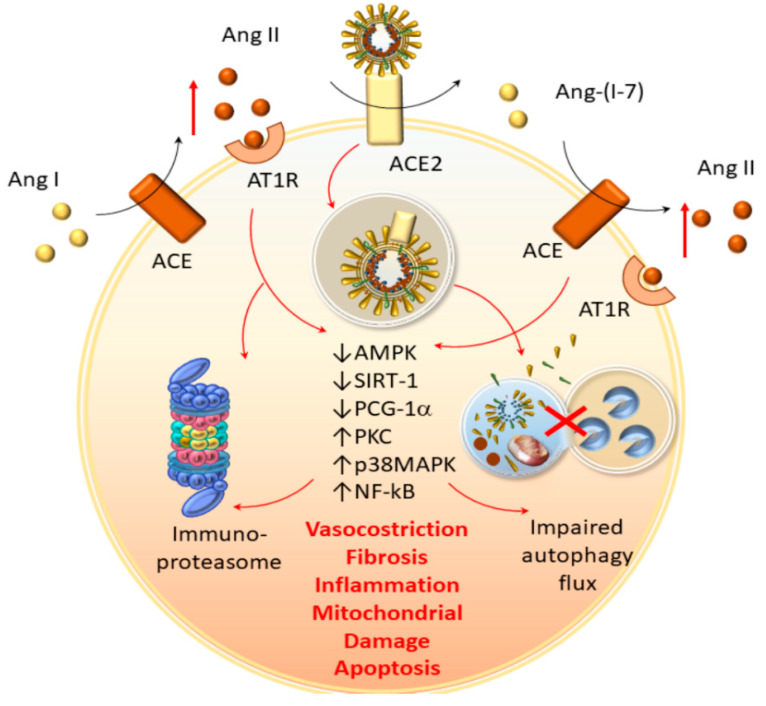
ACE2 degrades angiotensin II (Ang II, vasoconstrictor) to angiotensin 1–7 (Ang-1-7, vasodilator), while ACE promotes the synthesis of Ang II. Increased levels of Ang II occur following ACE2-SARS-CoV-2 binding and ACE2 endocytic internalization. This leads to a reduction of ACE2 available on the membrane surface and abnormal activation of the Ang-II/AT1R axis, which occludes the protective effects of ACE2, thus, promoting vasoconstriction, fibrosis, inflammation, mitochondrial damage, and apoptosis, meanwhile recruiting the immunoproteasome, and impairing autophagy.

Remarkably, the immunoproteasome-dependent editing and loading of viral antigens on MHC-I molecules occur in cooperation with the ACE enzyme, which exerts opposite effects to those of ACE2 [144]. Contrary to ACE2 which degrades angiotensin II (Ang II, vasoconstrictor) to angiotensin 1–7 (vasodilator), ACE promotes the synthesis of Ang II while adding to the immunoproteasome-dependent generation of MHC-I peptides [144]. Ang II, while acting as a vasoconstrictor through the AT1 receptor (AT1R), promotes immunoproteasome activation in various experimental models [131,132,145,146,147,148]. The synergistic interaction between ACE, Ang-II, and the immunoproteasome might be key, since, despite ACE2 fostering intracellular SARS-CoV-2 entry, the endocytic internalization of ACE2-SARS-CoV-2 complexes might reduce the amount of ACE2 available on the membrane surface, leading to vasoconstriction [149,150]. This is bound to abnormal activation of the Ang-II/AT1R axis, which recruits the immunoproteasome, and occludes the protective effects of ACE2 against acute lung injury, cardiovascular damage, and neurogenic hypertension [131,132,145,146,147,148,150,151]. Remarkably, while autophagy inhibition might exacerbate the effects of ACE2 loss- and Ang II-induced pro-inflammatory responses and mitochondrial alterations [130,152,153], immunoproteasome inhibition rescues autophagy and ameliorates Ang II-related hypertension, cardiac hypertrophy, and myocarditis [126,131,132,145,146,147,148,154]. These pieces of evidence support the opposite role of autophagy and immunoproteasome in COVID-19-related multisystem complications, which remains to be experimentally confirmed (Figure 4).

### 2.3. Autophagy Impairment, Exosome Release, and Inflammation

Autophagy impairment is known to trigger unconventional pathways aimed at getting rid of overwhelming intracellular cargoes through the release of either free or exosome-engulfed material [151]. Exosomes derive from intraluminal vesicles that are generated in the MVB compartment, following late endosomal membrane inward budding. MVBs can either fuse with autophagosomes and lysosomes where cargo degradation eventually occurs, or can fuse with the plasma membrane to release exosomes into the extracellular space [155]. On the one hand, this might be considered as a natural mechanism that cells have preserved to communicate with each other and share essential cell constituents within a common environment. On the other hand, when suppression of autophagosome maturation or fusion with the lysosome occurs, inadequate digestion of intracellular cargoes might promote the exosomal release of potentially deleterious material [48,155]. Thus, promoting autophagy flux through the fusion of MVBs with autophagosomes and lysosomes might reduce exosome biogenesis and secretion [48,142,156,157].

This is crucial since the intrapulmonary spreading of respiratory viruses, including CoVs, might occur through exosomes containing viral particles and also the proteasome subunits, which might spread either closely or at distant sites through the blood-stream or body fluids [156,157]. Thus, the spreading of SARS-CoV-2 particles might further occlude the autophagy machinery to produce severe inflammation in the wall of blood vessels, leading to vasculitis, which mainly affects highly perfused organs. This is supported by evidence showing that autophagy impairment might increase vascular inflammation, platelet aggregation, and atherosclerosis, which can be healed by autophagy activation [68,158]. Again, activation of autophagy is a beneficial process during cardiovascular damage induced by mechanical overload, ischemia, and oxidative stress in various animal models, where it ensures the preservation of ATP content and degradation of damaged mitochondria [45,133,159,160,161,162]. Autophagy activation is also beneficial in models of metabolic syndrome and diabetes [161,163], which are frequently associated with atherosclerosis and ischemic heart disease and represent an important risk factor for developing severe and critical forms of COVID-19 [164].

Exosome-based virus spreading might also recruit peripheral nerves as a gateway to enter the CNS. Such involvement might go beyond neuro-vasculitis to recruit in a site-specific fashion those nuclei that receive projection from viral-exposed organs. As reported for SARS-CoV, the vagal innervation of the lung might spread the virus within cardiorespiratory target nuclei of the brainstem [25], such as the dorsal vagal nucleus, the ala cinerea, the area postrema forming the dorsal respiratory group, as well as the interconnected ventral respiratory group where the pre-Boetzinger complex is placed. This engagement is consistent with the high expression of ACE2 mRNA and protein levels in the nucleus of tractus solitarius/dorsal motor nucleus of the vagus and the ventrolateral medulla [27]. ACE2 expression in brain areas involved in the control of cardiovascular function suggests that the carboxypeptidase might play a role in the central regulation of blood pressure and diseases involving the autonomic nervous system, such as hypertension. This might explain the abnormal cardiorespiratory activity adding to the peripheral involvement of the blood vessels in the lung and the heart, eventually contributing to cardiopulmonary fatalities occurring in COVID-19. Similarly, olfactory-related brain structures might be recruited when the virus progresses through the olfactory nerve to infect and promote the degeneration of neurons in the olfactory bulb and piriform cortex, as documented in SARS-CoV-infected mice expressing the receptor ACE2 [25]. Such a phenomenon might explain the typical anosmia that is documented in COVID-19 patients [165].

**Figure 5:**
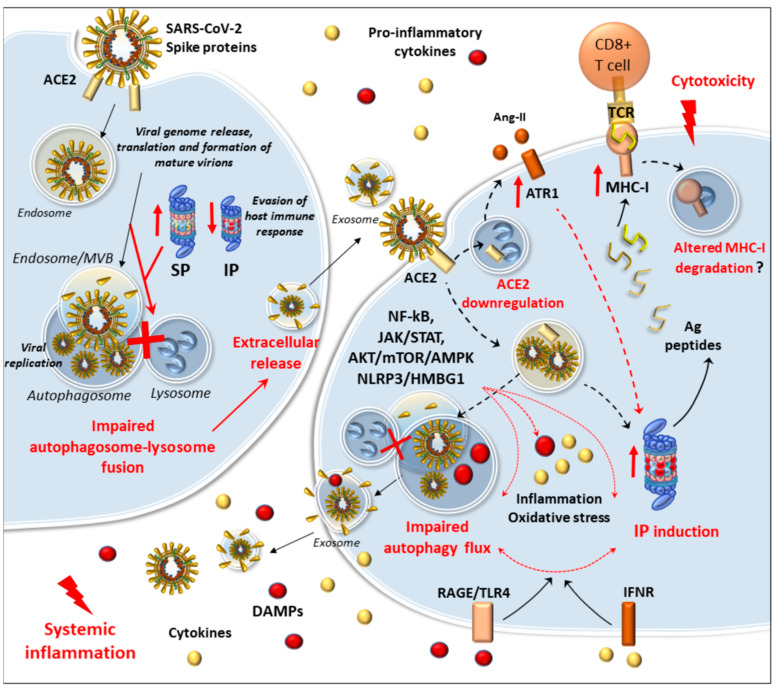
Summary of the potential mechanisms underlying SARS-CoV-2-induced alterations of autophagy and (immuno-)proteasome. SARS-CoV-2 is internalized within host cells (left side of the cartoon) upon the interaction of spike proteins with ACE2 and is first stored within the endosomal compartment. From here it releases the viral RNA upon membrane fusion to initiate the viral replication. Translation and eventual packaging of mature virions occur within the ER and Golgi (not shown). While increasing the number of autophagosomes to replicate herewith, SARS-CoV-2 blocks their fusion with lysosomes through a proteasome-dependent mechanism. This is likely due to the standard proteasome (SP) since immunoproteasome (IP) might be hijacked by the virus to avoid activation of the adaptive immune response. Impairment of autophagy flux eventually occludes the degradation of virions and viral components, while promoting their propagation from cell-to-cell, via exocytosis. Once released extracellularly, exosomes containing indigested viruses and viral material can reach distant tissues, besides neighboring cells (right side of the cartoon). SARS-CoV-2 also leads to ACE2 downregulation via AngII/AT1R-dependent internalization and lysosomal digestion. At the same time, a cascade of intracellular events (Ang-II-ATR1, PKC, NF-kB, JAK/STAT, AKT/AMPK/mTOR, NLRP/HMBG1) takes place to promote pro-inflammatory/oxidative events, while recruiting the immunoproteasome. These same events exacerbate the autophagy failure, which is induced by SARS-CoV-2, eventually promoting the extracellular release of virions and DAMPs along with activation of immunoproteasome-depended cytotoxic CD8+ T-cell response. In this way, DAMPs and cytokines further alter cell-clearing systems within host cells via binding to RAGEs, TLR4, and IFN receptors, while promoting systemic inflammation.

The extracellular spreading of the virus following impairment of autophagy–lysosome fusion is compatible with the trans-cellular diffusion of microvesicles that takes place in the CNS, to communicate physiological signals or spread disease through danger-associated molecular pathways (DAMPs). In fact, besides cell lysis, an autophagy impairment in virus-infected cells might promote the extracellular release of undigested, vesicular-packed DAMPs such as HMGB1 [166,167,168]. Once released extracellularly, HMGB1 initiates inflammation in either neighbor or distant host cells, via the activation of TLR4 and RAGE receptors, fuelling NLRP inflammasome activation and pro-inflammatory cytokine release [166,167]. These events might converge on further immunoproteasome recruitment and autophagy impairment in different kinds of cells, eventually promoting systemic inflammation and immune-mediated cytotoxicity [41,110,111,133,134,135,136,166]. This substantiates the hypothesis that excessive immunoproteasome recruitment going along with an autophagy impairment might contribute to sustaining the multisystem COVID-19 pathogenesis (Figure 5). Such a hypothesis is further supported by experimental evidence showing that the downregulation of immunoproteasomes, through activation of autophagy-related pathways, does counteract sepsis in animal models [69]. Additionally, this is supported by evidence from COVID-19 patients when focusing on CD8+ and CD4+ T-cell responses, which are bound to immunoproteasome- and autophagy-dependent antigen presentation, respectively. In fact, higher levels of CD8+ T-cells are detected in COVID-19 patients compared with controls, and a dramatic reduction of CD4+ T helper cells occurs in the most severe rather than mild COVID-19 cases [169].

Altogether, these pieces of evidence suggest that enhancing autophagy along with blunting immunoproteasome activity might be beneficial against excessive inflammatory conditions. In search of natural-based, potentially safe autophagy/(immuno-)proteasome modulators, in the following sections, we focus on some selected nutraceutical compounds that are known to provide antiviral and anti-inflammatory effects in the frame of respiratory, cardiovascular, and neuronal alterations, which might be relevant for COVID-19 multisystem pathogenesis.

## 3. Anti-Viral and Anti-Inflammatory Effects of Phytochemicals: Is There a Role for Cell-Clearing Systems?

In the context of COVID-19, phytochemical formulations and flavonoids from THM are now actively employed as an adjunct strategy to antiviral drugs [56,59,60,67]. Some encouraging results emerged for THM formulas that are rich in resveratrol, quercetin, and kaempferol [56,59,60,63,66]. This is documented in experimental models and patients affected by either sterile lung injury or respiratory viral infections, including SARS-CoV-2 [56,59,60,63,65,66]. This is also supported by network-based studies that identified resveratrol, quercetin, and kaempferol as “hub” components of THM formulas being employed in human respiratory viral infections, including influenza virus and SARS-CoV-2 [62,64]. These specific phytochemicals are predicted to exert the most effective pharmacological activity against influenza virus and SARS-CoV-2, by synergistically intervening in key pathways that are implicated in both viral replication and virus-induced inflammation, chemokine production, vascular permeability, and oxidative stress-induced apoptosis [62,64]. Similar mechanisms of action are reported for other less known, though potentially beneficial THM phytochemicals such as baicalein/baicalin, and cordycepin [57,61]. The list of nutraceuticals and herbal compounds that possess potential antiviral and anti-inflammatory activity is much longer. Here, we chose as an example only a few, specific, naturally-occurring compounds, to discuss evidence linking their mechanisms of action with autophagy and the (immuno)-proteasome. This might be relevant for various steps of SARS-CoV-2 infection, where autophagy and immunoproteasome are involved, ranging from viral replication to inflammation-related multisystem pathogenesis.

### 3.1. Resveratrol

As widely documented in the literature, the stilbene resveratrol induces autophagy and promotes autophagy flux through either AMPK/SIRT1 or TFEB activation, or PI3K/AKT/mTORC1/2 inhibition [68,161,162,170,171,172,173,174,175,176,177]. Besides induction of autophagy proteins such as LC3II, BECN1, Rab7, and ATG16L, potentiation of autophagy flux induced by resveratrol is evident by the enhanced clearance of autophagy–lysosomal substrates, including p62 and intracellular pathogens, and it is confirmed by the administration of autophagy/lysosome inhibitors, which indeed occlude the beneficial effects of resveratrol [68,161,170,171,172,173,174,175,176,177]. Resveratrol-induced autophagy is associated with anti-inflammatory, anti-oxidant, and anti-apoptotic effects in a variety of inflammatory-related conditions, including bacterial infection, sepsis, acute lung injury, platelet aggregation, pulmonary thrombosis, hypertension, vascular endothelial inflammation, myocardial and hepatic injury, arthritis, subarachnoid hemorrhage-induced neuronal injury, and toxicant-induced neuroinflammation [68,161,162,170,171,172,173,174,175,176,177]. Resveratrol-induced autophagy is also associated with the rescue of Nrf2, which is involved in mitophagy (the selective removal of senescent/dysfunctional mitochondria by autophagy) and mitochondrial biogenesis, protection from oxidative stress, and caspase-dependent apoptosis, as well as downregulation of the HMGB1/TLR4/MyD88/NF-κB pathway, NLRP3 inflammasome, and pro-inflammatory cytokines production [68,171,172,173,174,175,176,177].

Remarkably, resveratrol also blunts immunoproteasome activation, and this is associated with autophagy induction and protective effects in the experimental models of sepsis and cardiac hypertrophy [69,127]. In detail, resveratrol, through immunoproteasome downregulation, prevents immunoproteasome-dependent PTEN degradation to foster autophagy induction in vivo, while inhibiting the expression of NF-kB, NLRP inflammasome, and pro-inflammatory cytokines in vitro [69,127]. As shown in the LPS-treated monocytes and cells of sepsis patients, resveratrol acts mainly through downregulation of the LMP7 (B5i) immunoproteasome subunit, which is responsible for its enhanced chymotrypsin-like activity [69]. The efficacy of resveratrol is comparable to that of the selective LMP7 inhibitor ONX-0914, though its effects on immunoproteasome inhibition are milder and occur in the absence of toxicity, as compared to ONX-0914.

Resveratrol also possesses anti-viral effects, though direct evidence linking cell-clearing pathways with the anti-viral effects of resveratrol is missing so far. However, resveratrol, either in vitro or in vivo, counteracts replication of the influenza virus, SARS-CoV, or MERS-CoV, through inhibition of the p38MAPK and PI3K/AKT/mTOR pathways, which are involved in autophagy impairment, besides virus-induced apoptosis and inflammation [89,178,179,180,181]. The in vivo and in vitro anti-influenza viral effects of resveratrol are already achieved at low, non-toxic concentrations of 1 mg/kg/day for 7 days and 62.5 μM, respectively [179,181]. The antiviral efficacy of resveratrol is reduced when the cells are treated with neutralizing anti-IFN-β antibodies, suggesting that it acts synergistically with IFN-β, to inhibit influenza virus replication, while promoting the activation of the host immune response [180].

Noteworthy, a recent study indicated a key role for resveratrol against the SARS-CoV-2 spike protein and human ACE2 receptor complex affinity and stability [182]. By using molecular dynamics simulation and binding free energy analysis based on molecular docking, resveratrol was found to rank first among those compounds showing the best affinity for spike protein—the ACE2 receptor complex. Thus, resveratrol holds potential as an anti-COVID-19 drug candidate, by acting through disruption of the spike protein—the ACE2 complex [182].

Some recent papers discussed evidence that resveratrol might provide beneficial multisystem effects in COVID-19 pathogenesis by rescuing downregulation of ACE2 and counteracting Ang II increase, which is expected to occur following viral infection [183,184]. This is associated with SIRT1-dependent downregulation of AT1R and Ang II levels [183,184]. In line with this, resveratrol also counteracts downstream events that are synergistically induced by Ang II and TNF-α in experimental acute pulmonary thromboembolism and pulmonary artery hypertension, including activation of p38MAPK, NF-kB, and monocyte chemoattractant protein-1 (MCP-1) [185,186]. Given the links among resveratrol, cell-clearing systems, and Ang II, it might be inferred that these effects are closely related to autophagy activation and immunoproteasome downregulation. However, this appears paradoxical when considering that ACE2 downregulation following SARS-CoV infection is bound to AT1R-induced ACE2 internalization and degradation into lysosomes, which does not support a beneficial role for autophagy inducers [147]. However, resveratrol might act at an earlier step of ACE2 internalization, either by blocking the SARS-CoV-2-spike protein—ACE2 receptor complex [182] or through Ang II-AT1R downregulation. This is expected to prevent ACE2 translocation to lysosomes while preserving both ACE2 and the autophagy activity. At the same time, resveratrol, through Ang II-AT1R downregulation, is supposed to blunt Ang II-related complications that are bound to immunoproteasome hyper-activation.

In line with this, resveratrol protects against experimental ALI, pulmonary embolism-induced cardiac injury, as well as cerebrovascular and neuronal inflammation, by mitigating signaling pathways that occur during viral infection and which are bound to a concomitant immunoproteasome activation and autophagy impairment, including RAGEs, HMGB1, TLR4, NF-kB, and NLRP3 [161,176,177,187,188]. Resveratrol-related inhibition of neuro-inflammation goes along with microglia polarization towards the anti-inflammatory M2 phenotype and it occurs through SIRT1 and PGC-1α activation [189,190], which are both bound to autophagy stimulation.

While experimental studies appear necessary to investigate whether these events occur in SARS-CoV-2, the evidence discussed here supports the hypothesis that resveratrol might play an active role against key events characterizing multisystem COVID-19 pathogenesis, at least in part, by targeting alterations in autophagy and immunoproteasome pathways.

### 3.2. Quercetin and Kaempferol

Both quercetin and kaempferol, two natural flavonoids, were shown to produce anti-inflammatory effects through autophagy induction [70,71,191,192]. In a mice model of severe progressive pulmonary fibrosis, kaempferol significantly inhibits pulmonary inflammation and inflammatory cells infiltration, through the restoration of LC3 lipidation and a concomitant increase in autophagy flux. In fact, blocking autophagy flux through autophagy/lysosome inhibitors antagonizes the anti-inflammatory effects of kaempferol during pulmonary fibrosis [70]. Again, kaempferol counteracts in vivo neuroinflammation and provides neuroprotection, by inhibiting NLRP3 inflammasome activation and IL-1β secretion [191]. These effects are associated with a rescue of autophagy and potentiation of autophagy flux in microglia, and subsequent degradation of ubiquitinated NLRP3, which is prevented by either ATG5 knockdown or treatment with autophagy/lysosome inhibitors [191].

Similarly, quercetin and its O-glycoside quercitrin are both able to induce autophagy producing anti-oxidant and anti-inflammatory effects, which are abolished by pharmacological autophagy inhibition in animal models of atherosclerosis [71,192]. Quercetin induces autophagy through mTOR inhibition, which is associated with an improvement of aortic ultrastructural morphology and decreased levels of TNF-α, IL-1β, IL-18 [71]. Quercetin, through autophagy induction, also inhibits NLRP3 activation while protecting mitochondrial integrity and inhibiting ROS production, upon bacterial infection in vitro [193]. Similar to resveratrol, quercetin activates the autophagy-related AMPK/SIRT1 axis to counteract HSV-1 neuro-infection, propagation, and subsequent increases in neuropathological protein aggregates [194]. As the main constituents of a hydroalcoholic extract from Fragaria vesca leaves, quercetin and kaempferol produce nitric oxide scavenging and anti-inflammatory activity in LPS-treated macrophages, which is associated with proteasome inhibition and autophagy induction [195].

This is in line with several studies suggesting that both quercetin and kaempferol, especially in their glycosylated forms, produce beneficial effects in a variety of conditions that might be relevant for COVID-19 multisystem pathogenesis, including lung inflammation and pulmonary fibrosis, coronary heart disease, myocardial infarction, diabetes, and neuroinflammation [70,196,197,198,199]. These effects are associated with a reduction of blood pressure, and inhibition of pro-inflammatory cytokine release, platelet aggregation, and ACE-Ang-II [198,199]. Neuroprotective effects of quercetin and kaempferol are widely documented in various experimental models, which are associated, either directly or indirectly, with modulation of autophagy and immunoproteasome pathways. These include reduction of oxidative stress and neuroinflammation, induction of M2 microglia polarization, improvement of BBB function, prevention of AGE/RAGE-related neuronal damage, inhibition of HMBG1/TLR4 axis, and activation of AMPK and Nrf2 [200,201,202,203,204,205,206]. Both quercetin and kaempferol, through inhibition of cytochrome P-450-dependent catalysis, prevent the hepatic metabolism of 17 beta-estradiol [207], whose effects are bound to autophagy activity [208] and are potentially relevant in the frame of either influenza virus or COVID-19 pathogenesis [209,210].

Remarkably, both quercetin and kaempferol possess anti-viral activity, although associations with cell-clearing systems are poorly investigated. Quercetin and kaempferol are among the most active antiviral components of *Aloe vera*, which effectively reduces the viral replication of the influenza virus in vitro, which is bound to the inhibition of autophagy [211]. In line with the binding affinity of quercetin and kaempferol for the M2 protein of the influenza virus, quercetin- and kaempferol derivatives were also identified as potential inhibitors of the 3C-like protease (3CL(pro)) of SARS-CoV [212] and SARS-CoV-2 [213]. Recent molecular binding studies suggest that both quercetin and kaempferol can block the interaction sites on the SARS-CoV-2 spike—ACE2 [214,215]. Quercetin and its derivatives quercetin-3-rhamnoside and isoquercetin, effectively inhibit the replication of the influenza virus both in vitro and in vivo [216,217,218]. In vitro, quercetin-3-rhamnoside inhibits the influenza virus replication with a higher efficiency, as compared with oseltamivir, though such an antiviral effect is potentiated when either quercetin, quercetin-3-rhamnoside, or isoquercetin are co-administered with oseltamivir [216,218]. Compared to other polyphenols that exert antiviral activity in vitro, isoquercetin shows the lowest values for the effective dose, for a 50% reduction on viral replication (ED50) and toxic dose for 50% cell death (TD50), namely 1.2 uM and 46 uM, respectively. During influenza virus infection, isoquercetin pre-administration at either 2 or 10 mg/kg/day decreases the influenza virus titers, pro-inflammatory markers, and pathological changes in the lung of infected mice [217]. Similar effects occur following pre-treatment with quercetin 20 mg/kg b.w., which reduces oxidative damage in the lungs and liver of infected mice [219]. In summary, quercetin and kaempferol counteract the replication of respiratory viruses and protect against viral-induced apoptosis, which warrants further studies investigating their potential efficacy in SARS-CoV-2 infection. This is also supported by the widespread, anti-inflammatory, and autophagy-related effects of quercetin and kaempferol in a variety of conditions that might be relevant for multisystem COVID-19 pathogenesis.

### 3.3. Cordycepin

Pioneering studies of the late 1970s’ showed that cordycepin, the main bioactive compound of the *Cordyceps* mushroom genus, selectively inhibits the replication of the influenza virus by occluding the intracellular viral RNA synthesis [220,221]. Since then, there is a paucity of studies on the effects of cordycepin against respiratory viruses, despite several ones documenting its antiviral activity in human immunodeficiency virus (HIV), murine leukemia virus, and Epstein-Barr virus (EBV) [222]. Nonetheless, cordycepin is widely used in the THM for the treatment of respiratory diseases and it appears beneficial in human lung diseases [57]. This is confirmed in animal models of chronic obstructive pulmonary disease, where cordycepin improves tissue morphological changes such as thinning of the airway walls, infiltration of inflammatory cells, and sub-epithelial fibrosis [223]. It also reduces the accumulation of macrophages, neutrophils, and lymphocytes in the bronchoalveolar fluid, while downregulating the inflammatory cytokines TNF-α, IL-8, TGF-β1 [223]. Similar effects are recapitulated through in vitro and in vivo in models of pulmonary inflammation and lung fibrosis, where cordycepin suppresses IL-1β and IL-18 secretion through inhibition of NLRP3 inflammasome, both in human-derived macrophages and in mice lung tissues [224,225]. At the same time, cordycepin restores superoxide dismutase (SOD) expression, while inhibiting ROS production in TGF-β1–treated lung fibroblasts and epithelial cells [225]. In LPS-activated macrophages and experimental ALI mice, cordycepin inhibits NF-kB, while reducing the expression of COX2, TNF-α, IL-6, and IL-1β [226,227]. This is associated with protection against alveolar epithelium damage and edema, and reduced infiltration of inflammatory cells in the bronchoalveolar fluid of ALI mice [227]. Remarkably, cordycepin was shown to induce autophagy and promote autophagy flux, as evident by the upregulation of BECN1 and LC3-II and downregulation of p62, which is associated with beneficial effects in experimental mice of diabetic nephropathy [72]. While reversing histopathological injuries in the kidney of diseased mice, cordycepin suppresses inflammation, apoptosis, and renal fibrosis by decreasing IL-1β, IL-6, and IL-18, as well as TUNEL staining, and the expression of fibrosis markers [72]. All these effects are abolished by the autophagy inhibitor 3-MA [72]. This is recapitulated in salt-sensitive rats that are fed via a high-salt diet, where cordycepin-induced autophagy is associated with lifespan extension and protection of hypertension-sensitive organs, including the brain, heart, kidney, and liver [228]. Morphologically, neurons, cardiomyocytes, glomerular podocytes, renal epithelial cells, and hepatocytes are all improved in cordycepin-treated rats featuring reduced levels of AKT/mTOR, increased levels of AMPK, and decreased levels of p62 witnessing for autophagy flux progression, as compared to the untreated controls [228]. A cordycepin derivative, through AMPK activation, also alleviates atherosclerosis in High-Fat Diet-Fed ApoE-KO mice, by protecting the vascular endothelial cells from inflammatory and oxidative damage [229]. Thus, cordycepin might be a potential modulator of autophagy to be tested in the frame of SARS-CoV-2 infection.

### 3.4. Baicalein and Baicalin

The two major flavonoids of *Scutellaria baicalensis*, baicalein and its aglycone metabolite baicalin, possess widespread biological activities, including anti-bacterial, anti-viral, anti-inflammatory, anti-oxidant, neurotrophic, and neuroprotective effects [67,230]. Baicalein and baicalin show inhibitory effects on various strains of influenza virus and SARS-CoV, both in vitro and in vivo. Oral administration of baicalein to mice infected with the influenza virus increases the mean time to death, inhibits lung inflammation, and reduces the lung virus titer in a dose-dependent manner [231]. These effects are likely due to baicalin, the metabolite of baicalein, which was shown to inhibit both influenza virus and SARS-CoV replication in vitro [231,232]. In detail, baicalin showed in vitro antiviral activity against nine strains of SARS-CoV collected from patients’ lung tissue biopsy, urine, and nasopharyngeal aspirates [232]. The inhibitory effects of baicalin are similar to those of leukocytic IFN-α, IFN-β-1a, ribavirin, lopinavir, and rimantadine, though some differences in terms of efficacy are observed, depending on the cell lines employed [232]. The range of the effective concentration of baicalin required to reduce the plaque-forming unit (cytopathic effect) by 50% (EC50) was estimated as 11 μg/mL, and the cytotoxic concentration that reduced cell viability to 50% (CC50) was >100 μg/mL [232].

Baicalin was shown to inhibit influenza virus replication through TRAF6-dependent expression of Type-I IFNs, which correlated with protection against ALI in infected mice [233]. This is interesting since, in SARS-CoV, similar to what was observed in the influenza virus, inhibition of TRAF6-dependent expression of Type-I IFNs was associated with an abnormal UPS-dependent degradation, eventually leading to alterations in mitochondrial homeostasis and autophagy [234]. This fits with evidence documenting an inhibitory effect of baicalin on the chymotrypsin-like activity of the proteasome [73].

Remarkably, baicalin, sodium baicalin, and baicalein counteract the influenza virus infection, both in vitro and in vivo, by directly inhibiting the neuraminidase surface glycoprotein that is necessary for viral replication and the release of virions from infected cells [235,236]. Sodium baicalin is also effective against oseltamivir-resistant mutant influenza virus strains [236] while baicalein enhances the anti-viral effects of the neuraminidase inhibitor zanamivir [237]. The anti-neuraminidase activity of baicalein is accompanied by a downregulation of TNF-α, IL-6, and IL-8, which is associated with inhibition of the NF-kB and PI3K/AKT pathways, suggesting a potential activation of autophagy [237]. This is in contrast to studies associating the antiviral activity of baicalin with an attenuation of influenza virus-induced autophagy [238]. Such controversy might be related to either a viral-strain specificity or to the same paradoxical mTOR-related effects that are observed during SARS-CoV-2 infection [31]. Nevertheless, this calls for considering potential misinterpretations of the autophagy status since influenza virus was confirmed to block autophagy–lysosome fusion similar to CoVs [86]. This also fits with evidence showing that baicalin directly targets the NS1 protein of the influenza virus [239], which is shown to impair autophagy through PI3K/AKT activation [85,88]. Remarkably, baicalein can bind the N-terminus and C-terminus of the homology model of the SARS-CoV-2 proteins Nsp14 and 3CLpro, providing a potential candidate drug against SARS-CoV-2, for further study [240,241].

In addition to viral infections, baicalin and baicalein produce antioxidant and anti-inflammatory effects in several experimental inflammatory conditions, including diabetes, atherosclerosis, cardiovascular diseases, inflammatory bowel disease, rheumatoid arthritis, as well as liver-, kidney-, psychiatric- and neurodegenerative- diseases [67,242,243]. This is largely bound to key molecular pathways being implicated in the same antiviral activity of these compounds, namely attenuation of the NF-kB pathway and pro-inflammatory cytokines and chemokines such as TNF-α, IL-6 and IL-8, and MCP-1, ROS scavenging, and improvement of antioxidant status, as well as inhibition of COX2 and lipoxygenases [243]. The anti-inflammatory and anti-apoptotic effects of baicalin/baicalin are also bound to autophagy stimulation. In a model of arthritis consisting of IL-1β-treated chondrocytes, baicalin rescues autophagy to confer protection through the up-regulation of BECN1 and LC3 II/LC3 I ratio, and potentiation of autophagy flux, as evident by the decreased p62 levels [244]. In experimental models of hyperglycemia-induced myocardial damage, autophagy flux inhibitors occlude the anti-oxidant and anti-apoptotic effects of baicalin [245]. These findings are in line with evidence on baicalein acting as an inducer of autophagy flux, as documented by luciferase-based reporter assays [246].

Thus, baicalin and baicalein represent potential modulators of cell-clearing systems that deserve to be investigated in the frame of the multisystem COVID-19 pathogenesis. A summary of the anti-inflammatory, anti-viral, anti-apoptotic, and cell-clearing-related effects of phytochemicals discussed so far is provided in Table 1.

## 4. Conclusions

The current understanding of COVID-19 pathogenesis suggests uncontrolled host immune response and cytokine storm, though randomized trials providing evidence for any effective therapies against the disease are still lacking [249,250]. The absence of evidence-based medicine and approved drugs has shifted the focus on clinical insights and patient management through collaborative efforts in hospitals around the globe [249]. In the present review, we discussed evidence supporting the hypothesis that COVID-19 multisystem pathogenesis is bound to alterations of cell-clearing pathways within a variety of potentially affected tissues, including lungs, blood vessels, heart, and brain. The molecular hypothesis is grounded on data showing how viral particles within infected cells are entrapped within abundant autophagy-like vacuoles, which are actually impaired to merge with the lysosomes, thus, altering the clearance of the virus itself. Coupled with the spreading of pro-inflammatory reactions, this leads to a further autophagy impairment, while persistently recruiting the immunoproteasome. These events converge in spreading the virus to neighboring and distant sites on the one hand, while perpetuating systemic pro-inflammatory and cytotoxic immune reactions, on the other. Despite the significance of such an alteration still being unclear in the context of COVID-19, it seems to take place in each kind of affected/infected cell. In fact, it might explain the significance of myocardial alterations as well as the damage to blood vessels and neurons, where autophagy is critical to promote cell survival and modulation of inflammatory phenomena. In this scenario, it is fascinating how concepts from immune-hematology invading neuroscience might contribute to dissecting the role of cell-clearing systems during COVID-19 pathogenesis. Just like that described for viral infections, both autophagy and proteasome are key to prevent the accumulation and propagation of prionoids, such as alpha-synuclein, in either the CNS milieu or distant organs [251], while the immunoproteasome recruited under pro-inflammatory conditions cleaves alpha-synuclein, specifically within antigenic sites [141]. While serving as an alarmin for immune defense recruitment, this makes neurons and glia susceptible to cytotoxic immune attack [141]. Similar to NLRP/HMGB1, which propagates following an autophagy impairment during viral infections, prionoids and AGEs behave as DAMPs to trigger inflammatory reactions within host cells, via RAGEs and TLRs [48].

These concepts might also explain the widespread beneficial effects of a variety of phytochemical compounds that enhance autophagy, while blunting the (immuno-)proteasome. When focusing individually on each compound, shared molecular targets emerge bridging the pathogenesis of inflammation with autophagy and immunoproteasome alterations, including p38MAPK, PTEN/AKT/mTOR, NF-kB, and HMGB1/NLPR/TLR/RAGE axis. This might support the potentially synergistic, beneficial effects of phytochemical-rich formulations in multisystem viral infections involving the respiratory, cardiovascular, and nervous system, where the cell-clearing pathways are ubiquitously altered. Specific phytochemical-induced molecular pathways implicated in autophagy activation are also involved in blunting immunoproteasome activity. This suggests that the interplay occurring between autophagy and UPS/immunoproteasome also deserves to be investigated in the light of their contribution to pneumonia, ALI/ARDS, myocarditis, vasculitis, and neuro-inflammation, which take place during COVID-19. Despite COVID-19 representing a novel scenario that remains to be fully characterized from a molecular viewpoint, the findings here discussed suggest that polyphenol-rich formulations might counteract viral infections by promoting anti-inflammatory and immuno-modulatory effects that are bound, at least in part, to the tuning of autophagy and proteasome pathways. in addition to the specific compounds reviewed here, a plethora of additional herbal and nutraceutical compounds show anti-viral and widespread anti-inflammatory/anti-oxidant effects that are associated with cell-clearing systems [31,86,252,253]. These include, among others, spermidine, curcumin, epigallocatechin-gallate, melatonin, eugenol, and vitamin D, which similarly deserve to be investigated. Experimental and clinical studies subjected to rigorous scientific scrutiny are needed to confirm whether these phytochemicals might provide prophylactic or adjunct therapeutic support in COVID-19 pathogenesis, potentially by acting as natural-based, non-toxic autophagy/UPS modulators.

## Figures and Tables

**Table 1:** Summary of the anti-inflammatory, anti-viral, anti-apoptotic, and cell-clearing-related effects of phytochemicals.

Phytochemical and Experimental Model	Autophagy-Related Effects	UPS-Related Effects	Anti-Inflammatory Effects	Anti-Viral Effects	Anti-Apoptotic Effects
**Resveratrol**					
TNF-α-induced endothelial inflammation, and endothelial oxidative injury [68,171,247] LPS-treated monocytes and microglia [69,190] Cardiac hypertrophy/ Ang-II-treated cardiomyocytes [127] Diabetic cardiomyopathy [161] Intracellular bacterial infection [170] Monocytes from chronic kidney disease (CKD) patients [173] Pulmonary thrombosis, pulmonary artery hypertension, and platelet aggregation [174,185,186] Subarachnoid hemorrhage, hypoxic/ischemia brain injury, and spinal cord injury [175,177,248] Plasma cells from gout patients [176] AGEs-induced inflammation [188]	↑LC3II/LC3I ratio ↑BECN1 ↑Rab7 ↑ATG16L1 ↑PTEN ↑SIRT1/AMPK ↑TFEB ↑flux ↓p62 ↑xenophagy [68,127,161,170,171,173,174,175,176,247,248]	↓LMP7, LMP2 immunoproteasome [69,127]	↓ICAM-1 ↓NF-κB ↓IL-1β, IL-6, IL-8 ↓TNF-α ↓IFN-γ ↓iNOS ↓COX-2 ↓NLRP3 ↓MCP-1 ↓TLR4/MyD88/NF-κB ↓HMBG1 release ↓RAGE/MAPK/ NF-κB [68,69,170,171,173,175,176,177,185,186,188,190]	Influenza virus [179,180] MERS-CoV [181] ↓ACE2-SARS-CoV-2 S protein binding [182] HSV-1 [194]	↓caspase-3/9/12 ↓BAX/Bcl-2 ↓TUNEL ↓p-p38 MPAK [127,129,185,186,188,248]
**Quercetin and derivatives**					
Pulmonary fibrosis [70] Oxidized low-density lipoprotein-treated endothelial cells [192] Intracellular bacterial infection [193] Viral neuro-infection [194] LPS-triggered macrophages [195] Lung hypoxia [196] LPS-induced neuroinflammation [204] Spinal cord injury [205]	↑LC3II/LC3I ratio ↑BECN1 ↓Akt ↑AMPK/Sirt1 ↑flux [70,192,193,194,195]	↓Chymotrypsin-like activity [195]	↓IL-1β, IL-6, IL-8, IL-18 ↓TNF-α ↓NLRP3 ↓iNOS ↓COX-2 ↓STAT1/NF-κB [70,193,195,196,204,205]	Herpes simplex virus type-1 (HSV-1) [194] Influenza virus [211,216,217,218,219] ↑Interaction on SARS-CoV-2 and SARS-CoV proteins [212,213,214] ↓ACE2-SARS-CoV-2 S protein binding [215]	↓caspase-3 ↓BAX/Bcl-2 ↓TUNEL [192]
**Kaempferol**					
Atherosclerosis [71] LPS-triggered macrophages [195] LPS-, IFN-γ-, and SNCA-induced neuroinflammation and neurodegeneration [191,200,201,203]	↑LC3II/LC3I ratio ↑flux ↑AMPK/Nrf2 [71,191,195,201]	↓Chymotrypsin-like activity [195]	↓IL-1β, IL-18, IL-6, IL-8 ↓TNF-α ↓NLRP3 ↓iNOS ↓COX-2 ↓MCP-2 ↓TLR4/MyD88/NF-κB ↓HMBG1 release [71,191,195,200,201,203]	Influenza virus [211] ↑Interaction on SARS-CoV-2 proteins [214] ↓ACE2-SARS-CoV-2 S protein binding [215]	↓caspase-1 [191]
**Cordycepin**					
Diabetic nephropathy [72] Chronic obstructive pulmonary disease, pulmonary inflammation, and fibrosis [223,224,225] LPS-triggered macrophages [226] LPS-induced acute lung injury [227] Hypertension-induced multi-organ damage [228] Atherosclerosis [229]	↑LC3II/LC3I ratio ↑BECN1 ↑flux ↓p62 ↑AMPK ↑Cathepsin D ↑mitochondrial function/mitophagy [72,228,229]	-	↓TNF-α ↓TGF-β ↓ IL-1β, Il-6, IL-8, IL-18 ↓NLRP3 ↓NF-κB ↓iNOS ↓COX-2 [72,223,224,225,226,227,229]	Influenza virus [220] Epstein-Barr virus [222]	↓caspase-3 ↓BAX/Bcl-2 ↓TUNEL [72]
**Baicalin/baicalein**					
High glucose-induced vascular inflammation [242] Arthritis [243] Hyperglycemia-induced cardiovascular alterations [244]	↑LC3II/LC3I ratio ↑BECN1 ↑flux ↓p62 ↓PI3K/AKT [237,244,245]	↓Chymotrypsin-like activity [73]	↑Type I IFN [231] ↓ICAM-1 ↓NF-κB ↓IL-8 ↓MCP-1 [237,242]	Influenza virus [231,233,235,236,237,238,239] SARS-CoV [232] ↑Interaction on SARS-CoV-2 proteins [240,241]	↓caspase-3 ↓BAX/Bcl-2 ↓PI staining and flow cytometry [243,244]

## Abbreviations

3-MA	3-Methyladenine
ACE(2)	angiotensin-converting enzyme (type 2)
AGE	advanced glycation end products
AKT	protein kinase B
ALI	acute lung injury
AMBRA1	activating molecule in beclin-1 regulated autophagy
AMPK	5′ AMP-activated protein kinase
Ang II	angiotensin II
ARDS	acute respiratory distress syndrome
AT1R	angiotensin II receptor type 1
ATG1/ULK1	Unc-51 like autophagy activating kinase-1
BBB	blood-brain barrier
BECN1	beclin 1
CNS	central nervous system
COVID-19	coronavirus disease 2019
CoVs	coronaviruses
COX2	cyclooxygenase 2
DAMP	damage-associated molecular patterns
ER	endoplasmic reticulum
ERK/MAPK	extracellular signal-regulated kinase/mitogen-activated protein kinase
HMGB1	high mobility group box 1
IFN	interferon
IL	interleukine
JAK/STAT	janus kinase/signal transducer and activator of transcription
LMP7	low-molecular mass protein-7 also known as B5i
LPS	lipopolysaccharide
MCP-1	monocyte chemoattractant protein-1
MERS-CoV	middle east respiratory syndrome coronavirus
MHC-I/II	Major histocompatibility molecules class I/II
MODS	multiple organ dysfunction syndrome
mTORC1	mammalian target of rapamycin complex 1
MVBs	multivesicular bodies
NF-kB	nuclear factor kappa-light-chain-enhancer of activated B cells
NLRP3	NOD-, LRR- and pyrin domain-containing protein 3
Nrf2	nuclear factor erythroid 2-related factor 2
p38MAPK	p38 mitogen-activated protein kinase
PGC-1α	peroxisome proliferator-activated receptor gamma coactivator 1-alpha
PI3K	phosphatidylinositol 3-kinase
PKC	protein kinase C
PTEN	phosphatase and tensin homolog
Rab GTPases	G coupled Ras-related proteins in brain
RAGE	receptor for advanced glycated end products
RAS	renin-angiotensin-system
Rb	retinoblastoma
RhoA	ras homolog gene family member A
ROS	reactive oxygen species
SARS-CoV	severe acute respiratory syndrome coronavirus
SARS-CoV-2	severe acute respiratory syndrome coronavirus 2
SIRT1	sirtuin-1
SNARE	soluble N-ethylmaleimide-sensitive factor attachment protein receptor
SQSTM1/p62	sequestosome 1
STX17	syntaxin-17
TFEB	transcription factor EB
THM	traditional herbal medicine
TLR	toll-like receptor
TNF	tumor necrosis factor
TRAF6	TNF receptor-associated factor
UPS	ubiquitin-proteasome system
VPS34	vacuolar protein sorting 34
vRNP	viral ribonucleoprotein
